# Design and Analysis of Linear Fault-Tolerant Permanent-Magnet Vernier Machines

**DOI:** 10.1155/2014/483080

**Published:** 2014-03-26

**Authors:** Liang Xu, Jinghua Ji, Guohai Liu, Yi Du, Hu Liu

**Affiliations:** School of Electrical and Information Engineering, Jiangsu University, Zhenjiang 212013, China

## Abstract

This paper proposes a new linear fault-tolerant permanent-magnet (PM) vernier (LFTPMV) machine, which can offer high thrust by using the magnetic gear effect. Both PMs and windings of the proposed machine are on short mover, while the long stator is only manufactured from iron. Hence, the proposed machine is very suitable for long stroke system applications. The key of this machine is that the magnetizer splits the two movers with modular and complementary structures. Hence, the proposed machine offers improved symmetrical and sinusoidal back electromotive force waveform and reduced detent force. Furthermore, owing to the complementary structure, the proposed machine possesses favorable fault-tolerant capability, namely, independent phases. In particular, differing from the existing fault-tolerant machines, the proposed machine offers fault tolerance without sacrificing thrust density. This is because neither fault-tolerant teeth nor the flux-barriers are adopted. The electromagnetic characteristics of the proposed machine are analyzed using the time-stepping finite-element method, which verifies the effectiveness of the theoretical analysis.

## 1. Introduction

Currently, linear machines have been applied to numerous direct-drive applications such as urban rail transit. It is well known that linear induction machine (LIM) has been applied to the metro traction system in many cities. The linear machine possesses the advantages of the enhanced train climbing ability and the reduced train turning radius. This in turn means increased cornering ability and improved stability. However, the efficiency and the power factor of the LIM are relatively low [1]. In order to overcome these drawbacks, the research on linear permanent-magnet (PM) synchronous machine has been increasingly prompted. However, its cost becomes a challenging issue for long stroke applications, since PM or copper cost increases with the system length. To solve the cost issue, several primary-PM linear machines which are the counterpart of stator-PM rotary machines have been considered [2–6]. The common characteristic of the primary-PM linear machine is that both the PMs and windings are on the short primary mover. It has been identified that this topology can reduce the cost for long stroke system.

The linear flux-reversal PM (LFRPM) machine retains the advantage of the primary-PM linear machine which is being cost effective for long stroke applications [2]. However, the thrust density of the LFRPM machine is relatively low because the leakage flux of PMs is too large, hence reducing the effective flux density in air gap.

Another attempt is the linear flux-switching PM (LFSPM) machine. In recent years, the design procedure and optimization of the LFSPM machine have been investigated [5]. It has been identified that this machine has a high air gap flux density due to the flux concentration effect [2]. Hence, compared with the existing LFRPM machine, it can provide greater thrust density under the same condition. However, the need of a higher PM volume for the LFSPM machine leads to the drastic increase of manufacturing costs.

Recently, the concept of magnetic gear (MG) has been presented and analyzed [7–11]. By using the principle of flux field modulation, the vernier PM (VPM) machine can offer improved torque density [12–15]. Also, it has a simpler structure differing from MG machine. In addition, in order to enhance the reliability of the VPM machine, a new fault-tolerant VPM machine was proposed in [12]. Its fault-tolerant capability was improved by introducing the fault-tolerant teeth. However, its torque density has also been reduced due to the reduced slot area.

The purpose of this paper is to design and analyze a new linear fault-tolerant PM vernier (LFTPMV) machine, which combines the advantages of the fault-tolerant machine and vernier machine, thus offering high thrust and high fault-tolerant capability. Also, in order to obtain the high fault-tolerant capability, the proposed machine adopts a delicate complementary structure, rather than conventional fault-tolerant teeth or flux-barriers structure in [5, 12]. Hence, the desired fault-tolerant capability is obtained without resulting in the reduction of the thrust density. In addition, the detent force of the proposed machine also reduced due to the complementary structure. Thus, the thrust performance of the proposed machine is improved.

## 2. Topology and Operation Principle

**Figure 1:**
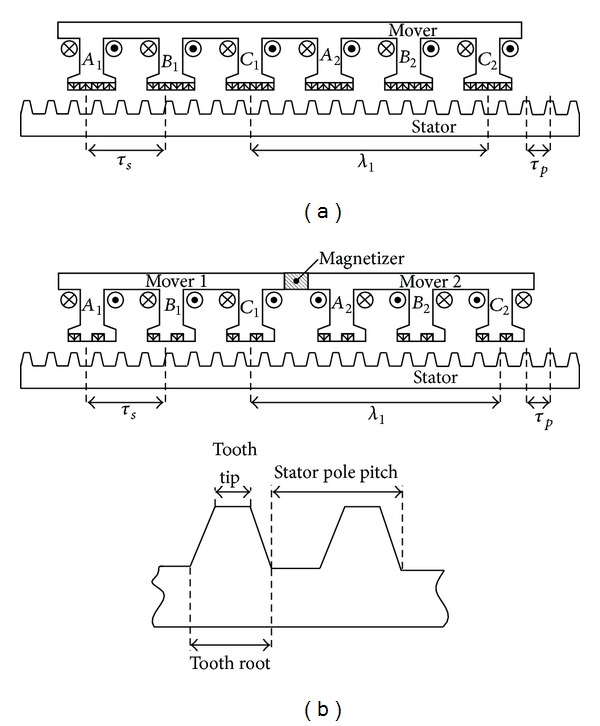
Comparison of topologies. (a) Existing LVH machine. (b) Proposed LFTPMV machine.

**Figure 2:**
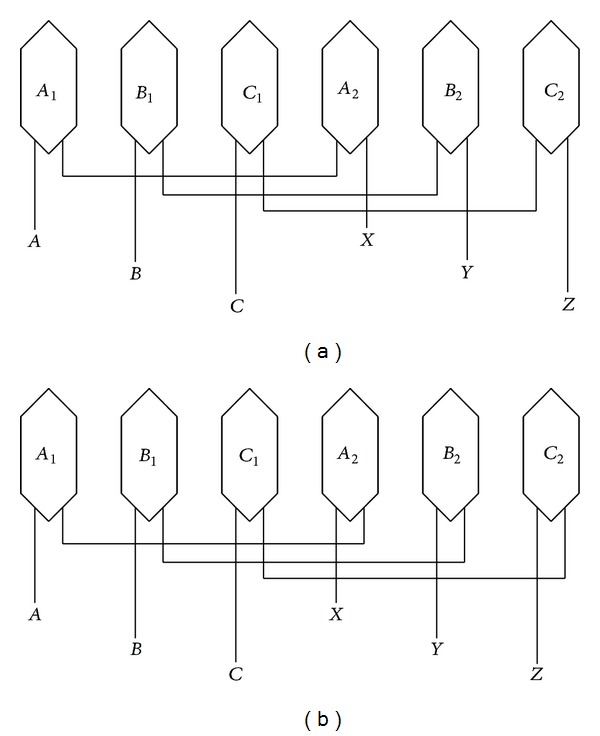
Schematics of armature windings. (a) Existing LVH machine. (b) Proposed LFTPMV machine.

Figure 1 compares the topologies of the existing linear vernier hybrid (LVH) machine and the proposed LFTPMV machine.  Figure 2 shows the schematics of their armature windings. It should be noted that the schematic of armature windings of the proposed machine significantly differs from that of the existing one. In the proposed machine, the armature windings of every phase are reversely connected in series. Phase *C*, for example, is composed of two concentrated armature windings, namely, *C*_1_ and *C*_2_. The positions of both windings are mutually *λ*_1_ = (*j* ± 1/2) × *τ*_*p*_ apart (*τ*_*p*_ is the stator pole pitch). Moreover, there is the magnetizer which connects the two adjacent mover modules. It should be noticed that the material of this magnetizer is the same as other parts of the mover. Thus, the magnetizer and the two mover modules are processed into a whole primary mover which can simplify the manufacturing process and assembly process. Between two adjacent phases belonging to one mover module, the distance equals to *τ*_*s*_ = (*k* ± 1/*m*) × *τ*_*p*_ (*k* = 3 is a positive integer and *m* = 3 is the phase number). The proposed machine adopts two PMs on the surface of each primary tooth. Compared with the existing one, the consumption of PM material can be reduced and PMs can be easily mounted. It also shows that the mover core of the proposed machine consists of two modules, named mover 1 and mover 2.

According to the operation principle of flux field modulation, the relationship among the overall PM pairsm *p*_PM_, the number of stator teeth *n*_*r*_, and the pole pairs of the spatial field distribution *p* is given by

(1) $$\begin{array}{c} n_{r}=p_{\text{PM}}\pm p. \end{array}$$

It should be noted that if the PMs do not entirely fill air gap along the level direction, the value of *p*_PM_ is not equal to the number of all existing PM pole pairs amounted on the iron. The existing LVH and proposed LFTPMV machines also operate according to the operation principle of MG. However, both of them have relatively large slot openings and there are no PMs in the slot openings which are not the same as the ones in [7–11]. Hence, (1) should be revised when it is used for the existing LVH and the proposed LFTPMV machines.

To analyze the two machines in this paper, the magnetic resistance and saturation of the steel are considered to be negligible. The effective magnetic motive force (MMF) produced by the PMs can be expressed as

(2) $$\begin{array}{c} F\left(x\right)=\overset{\infty }{\underset{m=1,3,5}{\sum }}F_{m}\cos\left(\frac{2\pi p_{{\text{PM}}_{\text{eff}}}x}{l}\right), \end{array}$$

where *x* is the mover position, *p*_PM_eff__ is the effective number of PM pole pairs on the primary mover, F_m_ is the amplitude of the *m* order component of MMF, and *l* is the primary length of the machine. Only considering the fundamental component, the effective MMF of the PMs can be simplified and expressed as

(3) $$\begin{array}{c} F\left(x\right)=F_{1}\cos\left(\frac{2\pi p_{{\text{PM}}_{\text{eff}}}x}{l}\right). \end{array}$$

Permeance coefficient changes with secondary moving and its toothed structure. Hence, both the moving velocity and corresponding secondary position influence the permeance coefficient. The permeance coefficient can be expressed as

(4) $$\begin{array}{c} P\left(x,t\right)=P_{0}+\overset{\infty }{\underset{i=1,2,3}{\sum }}P_{i}\cos\frac{2\pi n_{r}\left(x-vt\right)}{l}, \end{array}$$

where *P*_0_ is the direct current component in permeance coefficient, *P*_*i*_ is the amplitude of the *i* order component of permeance coefficient, *n*_*r*_ is the number of the stator teeth, and *v* is the secondary velocity. Only considering the fundamental component, the permeance coefficient can be simplified and expressed as

(5) $$\begin{array}{c} P\left(x,t\right)=P_{0}+P_{1}\cos\frac{2\pi n_{r}\left(x-vt\right)}{l}. \end{array}$$

The air gap magnetic flux density can be expressed as

(6) B(x,t)=F(x)P(x,t)=F1P0cos⁡2πpPMeffxl +12F1P1cos⁡2π(pPMeff+nr)x−2πnrvtl +12F1P1cos⁡2π(nr−pPMeff)x−2πnrvtl.

In (6), the first term represents a static magnetic field that cannot induce voltage in the primary windings. The second term represents short wavelength magnetic field produced by PMs and stator teeth, showing that the traveling speed of magnetic field is slow. The third term represents long wavelength magnetic field, showing that the traveling speed of magnetic field is fast that can induce more considerable voltage than the short wavelength magnetic field. Hence, the third term is chosen as to be effective harmonic to match with the pole pairs of primary windings, resulting in the following relationships:

(7) $$\begin{array}{c} p_{wi}=|n_{r}-p_{{\text{PM}}_{\text{eff}}}|, \end{array}$$

(8) $$\begin{array}{c} G_{r}=\frac{n_{r}}{p_{\text{wi}}}, \end{array}$$

(9) $$\begin{array}{c} v_{\text{flux}}=G_{r}v, \end{array}$$

where *p*_wi_ is the number of primary winding pole pairs, *G*_*r*_ is the gear ratio, and *v*_flux_ is the speed of the traveling magnetic field.

It can be found that (7) slightly differs from (1), because the primary slot openings of the proposed machines also influence the harmonic components of the PMs. Hence, the *p*_PM_eff__ must be confirmed and satisfied with (7) in order to use MG effect and obtain high thrust.

**Figure 3:**
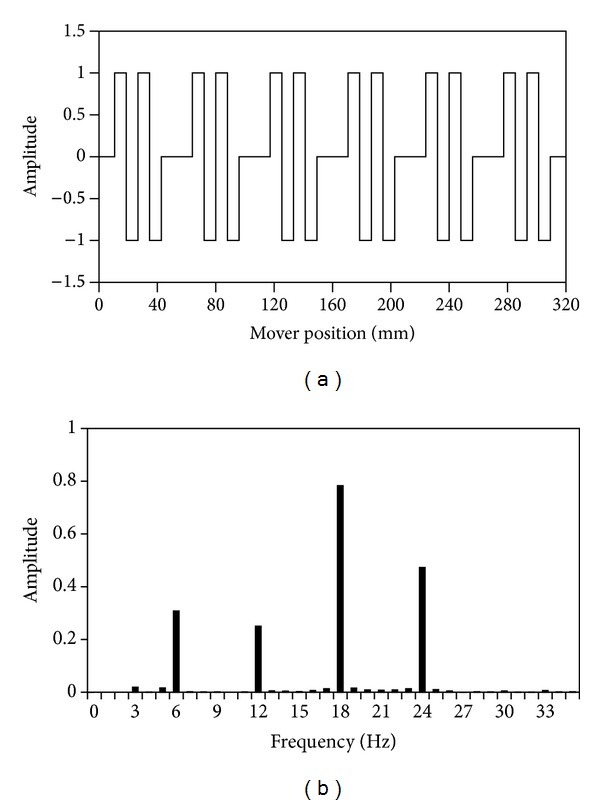
PM MMF waveform and spectra. (a) PM MMF waveform. (b) Spectra.

For the existing LVH machine, Figure 3 shows the PM MMF waveform and its spectra. It is noticed that 12, the number of the actual PM pole pairs, is not the highest amplitude, due to the primary slot openings. Also, it reveals that the 18-order harmonic is the main harmonic component. Therefore, the effective number of PMs is *p*_PM_eff__ = 18, and the number of stator teeth is *n*_*r*_ = 20. According to (7), the pole pairs produced by primary windings should be *p*_wi_ = 2.

## 3. Optimization

**Figure 4:**
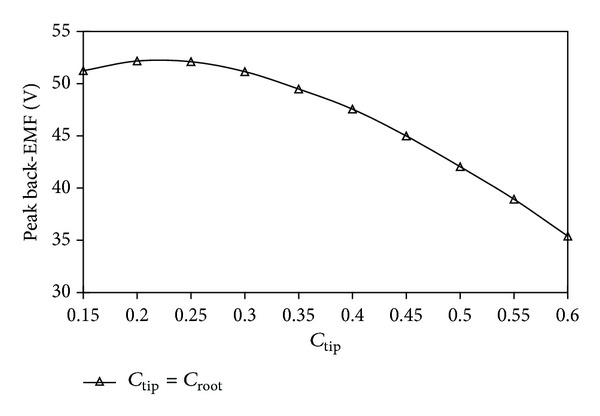
Peak value of back-EMF with respect to *c*_tip_.

**Figure 5:**
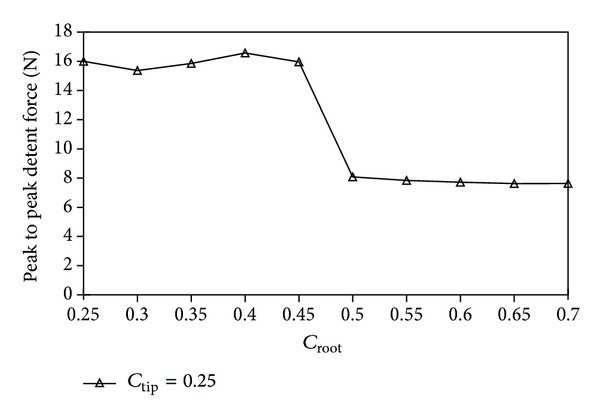
Peak to peak value of detent force with respect to *c*_root_.

The parameters of the stator tooth, such as *c*_tip_ and *c*_root_, can influence the performance characteristics of the proposed machine, where *c*_tip_ is defined as the ratio of stator tooth tip width to the stator pole pitch and *c*_root_ is defined as the ratio of stator tooth root width to the stator pole pitch as shown in Figure 1(b). In [16], the authors exhibited that stator tooth dimensions affect the average thrust of the machine. Also, the value of the *c*_root_ has negligible impact on the average thrust of the existing machine. In contrast, the value of the *c*_tip_ has significant impact on its average thrust. Based on these constraints, back electromotive force (back-EMF) optimization of the proposed machine can be simplified when the value of the *c*_root_ is defined the same as the value of the *c*_tip_.  Figure 4 shows the peak value of back-EMF of the proposed machine with respect to the value of the *c*_tip_. It can be observed that the peak value of back-EMF reaches maximum when the *c*_tip_ equals 0.2 or 0.25.  Figure 5 shows the peak to peak value variation of detent force of the proposed machine with respect to the *c*_root_ when the *c*_tip_ is chosen as 0.25. It can be known that the optimized *c*_root_ is from 0.5 to 0.7. To maximize peak back-EMF and minimize peak to peak detent force of the proposed machine, *c*_tip_ and *c*_root_ are chosen as 0.25 and 0.5, respectively.

## 4. Performance Analysis

### 4.1. Modular Structure

**Figure 6:**
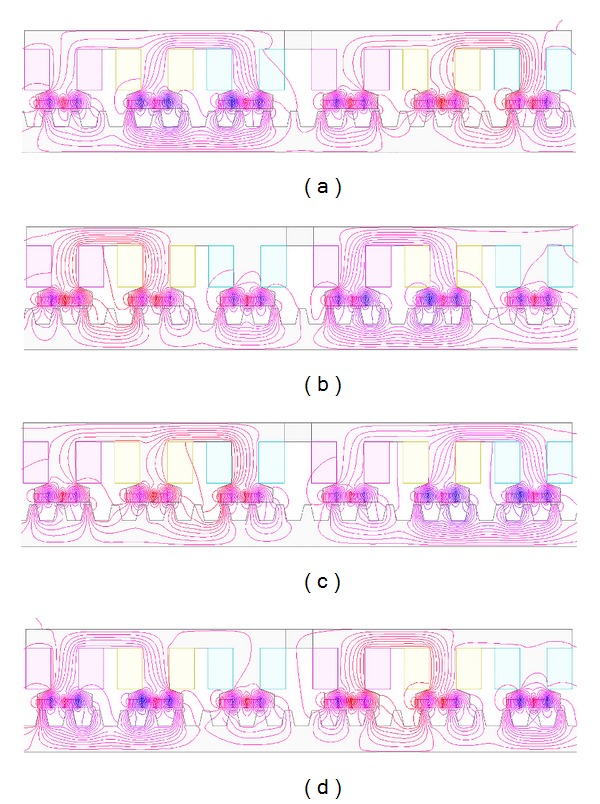
No-load magnetic field distributions at different stator positions. (a) Position* A*. (b) Position* B*. (c) Position* C*. (d) Position* D*.

The open-circuit field distributions under only PM excited are investigated based on finite-element method.  Figure 6 shows the no-load magnetic field distributions of the proposed machine at different mover positions. Position* A* is defined as the original mover position. Positions* B*,* C,* and* D* represent when the mover goes to the left from position* A* by 1/4, 1/2, and 3/4 stator pole pitches, respectively. The mover goes half stator pole pitch (180°) from position* A* to position* C*. It can be observed that PM flux distributions of position* A* in the mover 2 are very similar to those of position* C* in the mover 1. Also, PM flux distributions of position* B* in the mover 2 are very similar to those of position* D* in the mover 1. Therefore, it can be concluded that the change of flux distributions in both parts of the mover has difference in time for half of electrical period. Meanwhile, since there are very few flux lines which pass the magnetizer, the whole mover can be regarded as two independent modules with complementary magnetic circuit. Therefore, the proposed machine can be analyzed by one of both modules, rather than one whole module, which facilitates the interpretation of the MG effect in the proposed machine. Thus, for either of the two modules, the effective number of PMs is *p*_PM_eff__ = 9, the number of stator teeth is *n*_*r*_ = 10, and the pole pairs produced by primary windings are *p*_wi_ = 1. Obviously, they can satisfy (7).

**Figure 7:**
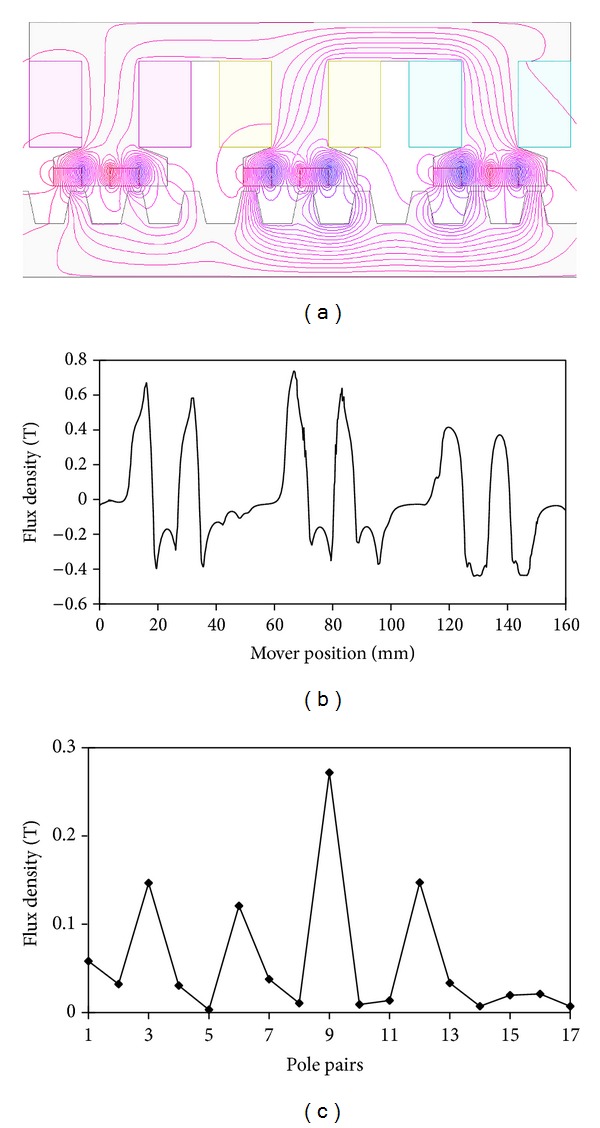
(a) No-load magnetic field distributions in the mover 1 at position* A*. (b) No-load air gap flux density. (c) Spectra.

Figure 7(a) shows the no-load magnetic field distributions in the mover 1 at position* A*.  Figure 7(b) shows the no-load flux radial density in the mover 1 at position* A*, which is modulated by the stator teeth. It is very crucial to analyze the no-load density in the air gap and its harmonics.  Figure 7(c) depicts the corresponding harmonic spectra. There are many asynchronous space harmonics in the air gap as the modulation of the stator teeth. Owing to the principle of flux field modulation (8), the fundamental flux travels at the speed 10 times of the speed of the secondary. Namely, *G*_*r*_ = 10.

### 4.2. Back-EMF

**Figure 8:**
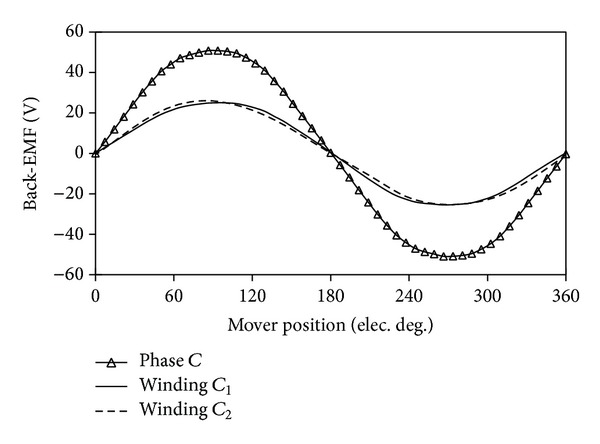
Winding and phase back-EMF waveforms.

**Figure 9:**
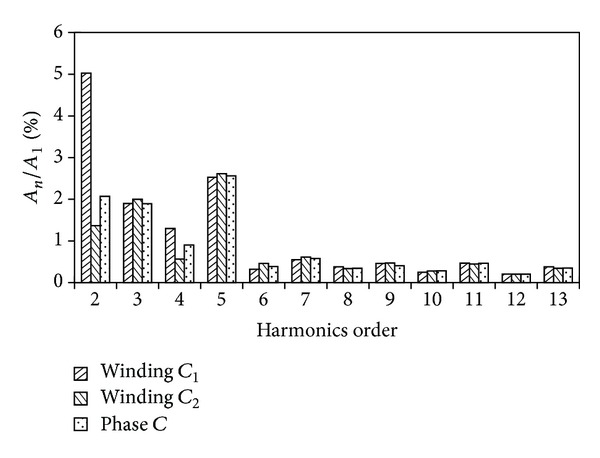
Harmonics analysis of winding and phase back-EMFs.

**Figure 10:**
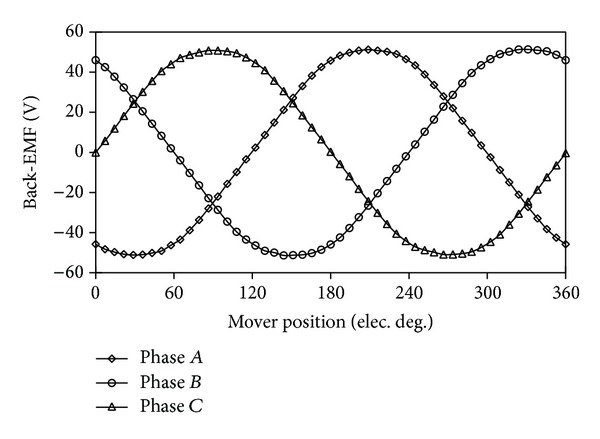
Three-phase back-EMF waveforms.

Figure 8 compares the back-EMF waveforms induced in winding* C*_1_, winding* C*_2,_ and phase* C* of the proposed machine at rated speed.  Figure 9 shows their harmonics analysis results. The back-EMF waveforms of phase* C* are the overlap of both winding* C*_1_ and winding* C*_2_. It can be seen that the even harmonic components of back-EMF greatly decrease, resulting in the phase back-EMF being more symmetrical and sinusoidal. This is due to the fact that the two modules of the proposed machines have the complementary magnetic circuits. Another reason is that the two concentrated armature windings belonging to one phase are reversely connected in series as is shown in Figure 2.  Figure 10 confirms that the three-phase back-EMF waveforms are very symmetrical and sinusoidal.

### 4.3. Detent Force

**Figure 11:**
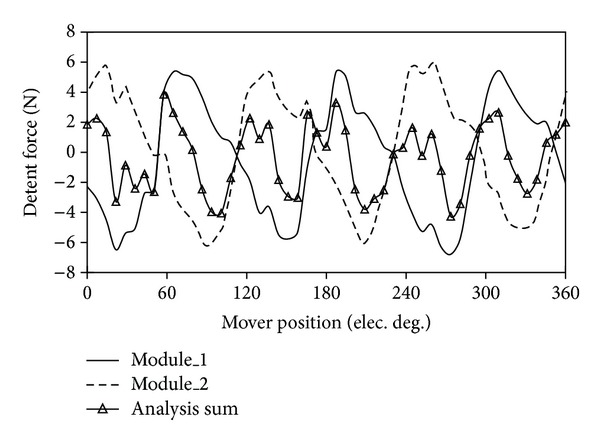
Partial and sum detent forces of proposed machine.

**Figure 12:**
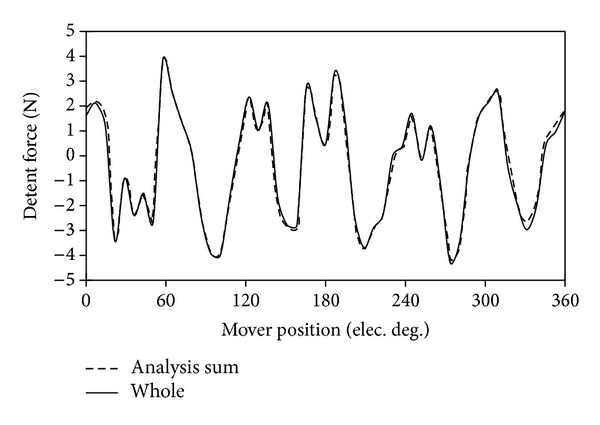
Comparison of detent forces based on both methods.

Figure 11 shows the detent force of the proposed machine. The detent force of the two modules of the proposed machine can be separately calculated by using FEM. In Figure 11, they are noted as “module_1” and “module_2.” As shown, the waveform of detent force of “module_2” is similar to the one of “module_1,” which shifts 180° along the horizontal axis. Hence, there are 180° phase angle differences between the two modules. The sum of “module_1” and “module_2” is defined as “analytical sum.” The “whole” means the total detent force, which is calculated directly by using FEM.  Figure 12 shows the waveforms of “analytical sum” and “whole.” It should be noticed that the waveform of “analytical sum” is very similar to the “whole” one.

### 4.4. Inductance

**Figure 13:**
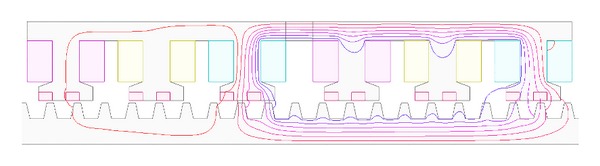
Magnetic field distributions only excited by armature current.

**Figure 14:**
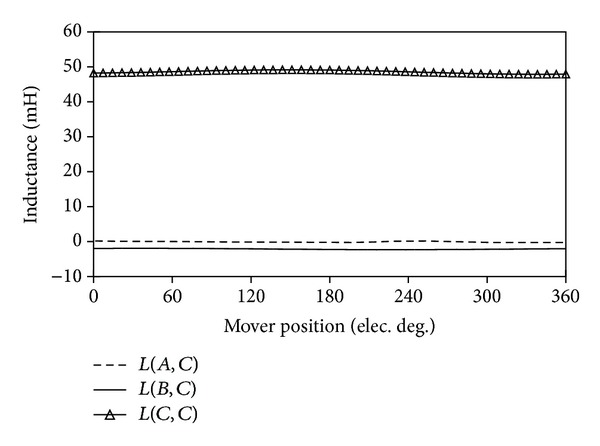
Comparison of inductances.

Figure 13 shows the flux pattern, in which all the PMs are unmagnetized and only phase* C* is excited. It can be seen that almost all of the produced fluxes pass the armature teeth of phase* C*, rather than other armature teeth. This means that the proposed machine decouples the phases.  Figure 14 compares the self-inductance and the mutual inductance of the proposed machine. It can be calculated that the ratio of the mutual inductance to the self-inductance of the proposed machine is only 4.4%. Hence, the proposed machine inherently possesses the desired fault-tolerant capability.

## 5. Comparison

In order to evaluate the proposed LFTPMV machine as compared with the existing LVH machine, they are designed based on the same number of phases, rated speed, stack length, winding turns, number of stator teeth, number of mover teeth, and stator pole pitch. According to the above-mentioned design procedure, a 3-pahse 6/20-pole LFTPMV and a 3-pahse 6/20-pole LVH machines are designed. Their major specifications are listed in Table 1.

The electromagnetic performances of the two machines are quantitatively analyzed using FEM. It can be found that the proposed machine requires 50% PMs of the existing one. Since PM material is much more costly, the proposed machine takes the significant advantage of lower cost.

Table 2 compares the self-inductance and the mutual inductance of the existing machine and the proposed machine. It illustrates that the self-inductance of the proposed machine is larger than that of the existing machine because the proposed machine has smaller total (magnetic) air gap length [17]. It can be calculated that the ratio of the mutual inductance to the self-inductance of the proposed LFTPMV machine is much lower than that of the existing LVH machine, revealing that each phase of the proposed machine is essentially decoupled from other phases. That is to say, the proposed machine possesses enhanced fault-tolerant capability.

**Figure 15:**
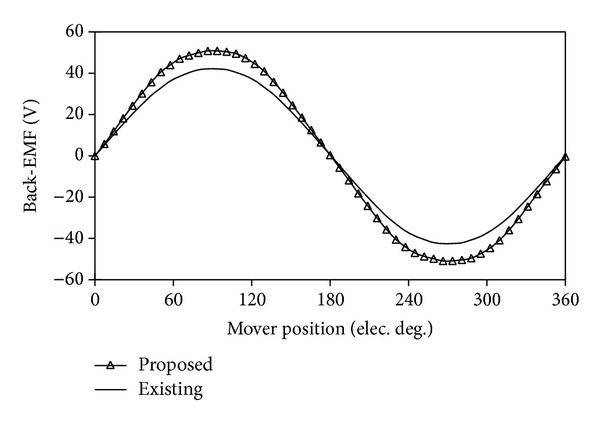
Comparison of back-EMF waveforms.

**Figure 16:**
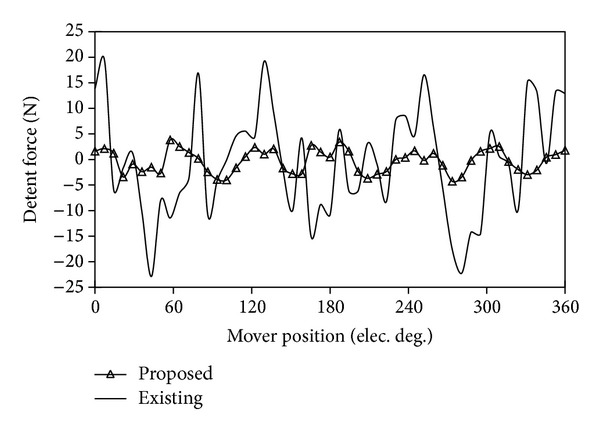
Comparison of detent force waveforms.

Figure 15 compares their back-EMF waveforms under the conditions of the same armature winding turns and speed. It can be found that the proposed machine generates higher back-EMF than the existing machine. Therefore, the thrust of the proposed machine is larger than that of the LVH machine when both machines operate at the same speed and electric loading.  Figure 16 compares their detent forces. It can be observed that the peak to peak value of the detent force of the proposed machine is much smaller than the one of the existing one.

**Figure 17:**
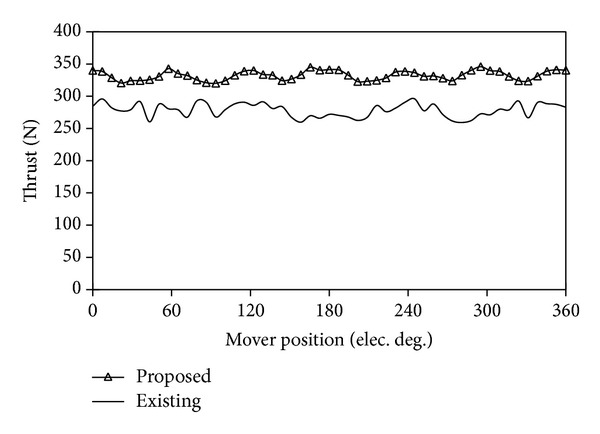
Comparison of thrusts.

Figure 17 shows the thrust waveforms of the proposed machine and the existing machine under brushless AC operation with the root mean square (RMS) value of 6 A. In order to evaluate the thrust performance of the two machines, a thrust fluctuation coefficient (*K*_*F*_) is defined as

(10) $$\begin{array}{c} K_{F}=\frac{F_{\max}-F_{\min}}{F_{\text{avg}}}=\frac{F_{\text{rip}}}{F_{\text{avg}}}, \end{array}$$

where *F*_max⁡_ is the maximum of thrust, *F*_min⁡_ is the minimum of thrust, *F*_avg_ is the average value of thrust, and *F*_rip_ is the value of thrust ripple. It can be calculated that the average thrust of the proposed machine is 332 N and the corresponding *K*_*F*_ is 7.8%, while the thrust and its ripple of the existing machine are 278 N and 13.3%, respectively. It indicates that the proposed machine possesses the better thrust performance.

## 6. Discussions and Conclusions

In this paper, a new LFTPMV machine has been proposed which is suitable for high thrust and long stroke applications. The parameters of the stator tooth, *c*_tip_ and *c*_root_, have been optimized for the maximal peak back-EMF and minimal detent force. However, the *p*_PM_ number and slot openings length also would have significant impacts on the magnetic loading and power density. Furthermore, for the proposed LFTPMV machine, its fault-tolerant capability would change with varied *p*_PM_ number and slot openings length. These issues are very critical for improved electromagnetic performance of the proposed machine. Therefore, it should be closely studied in the future work. On the other hand, since the ratio of the mutual inductance to the self-inductance of the proposed machine is very small, the proposed machine inherently possesses the desired fault-tolerant capability. Compared with the existing fault-tolerant machines, the proposed machine offers desired fault-tolerant capability without sacrificing thrust density because neither fault-tolerant teeth nor the flux-barriers are adopted. Due to the modular and complementary structure, the three-phase back-EMF waveforms are more symmetrical and sinusoidal. The detent force of the proposed LFTPMV machine is much smaller than that of the existing LVH machine. The proposed machine not only offers larger back-EMF but also reduces the cost of PMs. All of these advantages make the proposed machine an excellent candidate for high thrust and high reliability applications such as urban rail transit system.

## Figures and Tables

**Table 1:** Design parameters of existing LVH and proposed LFTPMV machines.

	Existing	Proposed
Number of phases	3	3
Rated speed (m/s)	1.6	1.6
Stack length (mm)	120	120
Mover length (mm)	320	328
Number of stator teeth	20	20
Winding turns per phase	232	232
Stator pole pitch (mm)	16	16
Air-gap length (mm)	1.5	1.5
Number of PMs	24	12
*C*_tip_	0.25	0.25
*C*_root_	0.5	0.5
PM material	NdFeB	NdFeB
Magnet remanence (T)	1.2	1.2
Magnet relative permeability	1.05	1.05
Magnet volume (cm^3^)	115.2	57.6
Mover/stator core material	DW465-50	DW465-50

**Table 2:** Comparison of self-inductance and mutual inductance.

	*L* (mH)	*M* (mH)	*M*/*L* (%)
Existing LVH machine	20.14	8.59	42.7
Proposed LFTPMV machine	48.61	2.13	4.4
